# Food as harm reduction during a drinking session: reducing the harm or normalising harmful use of alcohol? A qualitative comparative analysis of alcohol industry and non-alcohol industry-funded guidance

**DOI:** 10.1186/s12954-022-00648-y

**Published:** 2022-06-25

**Authors:** Anna Ramsbottom, May C. I. van Schalkwyk, Lauren Carters-White, Yasmine Benylles, Mark Petticrew

**Affiliations:** 1 London School of Hygiene and Tropical Medicine, 15-17 Tavistock Place, London, WC1H 9SH UK; 2 Old Medical School, Usher Institute, University of Edinburgh, Teviot Place, Edinburgh, EH8 9AG UK

**Keywords:** Alcohol, Alcohol industry, Discourse analysis, Public health, Commercial determinants of health

## Abstract

**Background:**

The aim of this study was to critically analyse information concerning the relationship between alcohol and food consumption provided via alcohol industry (AI) funded and non-AI-funded health-oriented websites, to determine the role it plays within the alcohol information space, and how this serves the interests of the disseminating organisations.

**Methods:**

Information on food as a harm reduction measure while drinking alcohol was extracted from 15 AI websites and websites of AI-funded corporate social responsibility (CSR) organisations. As a comparison group, non-AI-funded health websites were also searched (*n* = 16 websites with food and alcohol-related content). Information was included from webpage content and associated downloadable documents. Critical discourse analysis (CDA) was adopted to allow the texts analysed to be situated within the broader political and social context.

Analysis was carried out iteratively, involving continuous comparison within and between websites. Discursive themes were identified by three researchers. Identified discursive elements were discussed to reach a consensus, and a final coding framework was then developed. “Tone” analysis was used to assess whether the overall tone within individual websites was considered to be pro-alcohol consumption, neutral or discouraging of alcohol consumption.

**Results:**

There were some commonalities across AI and non-AI-funded websites, whereby both appeared to normalise alcohol consumption and to encourage use of food as a measure to enable sustained drinking, to avoid drinking in a way that results in short-term harms, and to prevent or “cure” a hangover. The fact that both AI-funded and non-AI-funded organisations shared many of these narratives is particularly concerning. However, a discourse of food and alcohol that served to promote “moderate” drinking as beneficial to health was used exclusively by AI-funded organisations, focusing on special occasions and individual-blaming.

**Conclusions:**

Alcohol consumption, including heavy and harmful consumption, is frequently normalised within the online information space. Emphasising food consumption with alcohol may have the effect of supporting consumers to drink for longer periods of time. Health professionals and independent health organisations should review the information they provide in light of our findings and challenge why AI-funded organisations, with a major conflict of interest, and a history of health misinformation, are often given the responsibility for disseminating health information to the public.

**Supplementary Information:**

The online version contains supplementary material available at 10.1186/s12954-022-00648-y.

## Background

Globally, alcohol use is the seventh leading risk factor for both deaths and disability adjusted life years (DALYs) [1]. Effective population-level alcohol harm reduction at a policy level focusses on interventions targeting the marketing, availability and pricing of alcohol. However, interventions implemented mainly focus on the provision of information, through labelling, and through education campaigns. The effectiveness of these is often limited, not least because labelling in many countries is self-regulated to some extend by the alcohol industry (AI) itself. The provision of information on alcohol harms also involves AI bodies, as well as independent public health actors, such as government agencies and NGOs. In the case of public health actors, the aim is to mediate the quantity and patterns of consumption that negatively affect health [2].

One harm reduction approach which is frequently recommended by both AI-funded and independent organisations is the consumption of food while drinking. Food consumption is explicitly mentioned in the low-risk alcohol consumption guidelines of several countries, with the aim of providing guidance on how to keep short-term risks low during single occasion drinking [3, 4]. However, the inclusion of such guidance varies and, for example, is absent from Australian or US guidelines [5–8]. Moreover, there is a lack of evidence presented within guidance documents to support the recommendation to consume food with alcohol for harm reduction purposes. This may reflect the extensive and heterogenous nature of the topic, or an absence of underlying evidence around the harm reduction potential of eating while consuming alcohol, though this is unclear. This in itself suggests that this is an appropriate topic for more detailed analysis. That these recommendations are widespread, yet these uncertainties remain, is a concern from a public health perspective and may undermine an evidence-informed approach to harm reduction.

The AI also disseminates information on this topic through corporate social responsibility (CSR) organisations which it funds (e.g. Drinkwise (Australia) and Drinkaware (Ireland and the UK)). This includes advice on eating in order to reduce harm during a drinking session. The accuracy of this information has not been assessed, though analysis of other health-related information funded by the AI has found that it misrepresents and omits evidence of harms in relation to cancers, cardiovascular disease (CVD), and the risk of harms in pregnancy [9–11]. There is also evidence that AI-funded organisations disseminate similar misinformation on alcohol harms and harm reduction to school children through school-based programmes in the UK and other countries [12].

The aim of this study was to critically analyse the discourses articulating eating as a form of alcohol harm reduction and to compare AI-funded and non-AI-funded materials. Our overall aims were to (i) analyse the available information on drinking and food consumption provided by AI-funded and non-AI-funded organisations and determine whether and how these discourses differ; and (ii) assess whether and how these discourses may establish and maintain systems of meaning and framing favourable to industry interests.

## Methods

### Data sources

AI-funded organisations predominantly disseminate information through industry websites (e.g. theheinekencompany.com) and the websites of CSR organisations which they fund (e.g. Drinkaware). We identified such organisations by firstly using previously published groups of organisations from two studies, the first by Lim et al. that similarly looked at information disseminated by AI and non-AI-funded organisations, specifically focusing on pregnancy, fertility and breastfeeding, and the second that analysed information on alcohol and cancer published by AI-funded organisations [9, 10]. These two studies had identified sources through searching the Global Alcohol Producers website and progress reports, and the CSR sections of alcohol producers’ websites. We carried out an updated search of the CSR sections in English language alcohol producers’ websites to identify any further relevant sources, published more recently. After excluding inaccessible websites and those without food and alcohol-related content, the websites of 15 industry-funded organisations were available for analysis. As a comparison group, non-AI-funded health websites were also searched (*n* = 16 websites with food and alcohol-related content) (see Table 1 for the full list of websites included). The independent (non-AI-funded) organisations included in this analysis were also based on those identified in the study by Lim et al., which had been compiled using a search of health websites of government bodies from the United States, United Kingdom, Canada, Australia, Ireland and New Zealand [10]. All information on food consumption as a harm reduction measure while drinking alcohol was extracted from both types of website. Information was included from both the webpages themselves and downloadable information sheets.

**Table 1:** AI-funded and non-AI-funded websites containing information on alcohol and food included in analysis

AI-funded (*N* = 15)	Non-AI-funded (*N* = 16)
Aware.org	Health.gov
Educ’alcool	MedlinePlus (U.S. national library of medicine)
Heineken: tips for drinking Responsibly	WebMD (US)
Foundation for advancing alcohol responsibility	NHS (NHS choices and www.nhs.uk)
DrinkWise (Australia)	CMO guidelines
Drinkaware (United Kingdom)	Alcohol focus (Scotland)
International alliance for responsible drinking	NHS inform Scotland
IARD education site, guidelines table	NHS direct wales
DrinkiQ (Diageo)	Northern Ireland direct
Bacardi website, "slow drinking"	Health service executive Ireland
Asahi website	department of health, Australia
Brown-Forman: our thinking about drinking	Queensland Government
Beam Suntory	Health direct Australia
Wine in moderation	Government of Canada health
AB InBev	Canadian centre of substance abuse and addiction: low risk drinking guidelines
	Ministry of health (NZ)

For information on a webpage to be eligible for data extraction, it was required to mention the consumption of food in relation to modifying the effects of alcohol consumption. The possible effects of alcohol are extensive and, as such, all types of specific health and health-related effects were considered relevant, including the health, social and behavioural effects. Information pertinent to the wider discourse of the subject was also extracted, for example information on the context and nature of “binge drinking” in relation to food, and the health risks associated with such behaviour. All relevant text was extracted into tables by one researcher, manually, with a second researcher independently checking the original data source for accuracy.

### Analysis methods

Critical discourse analysis (CDA) was used to allow the text analysed to be situated within the broader political and social context [13]. “Discourse” has contrasting definitions in the literature [14], and this study adopted Fairclough’s definition as “particular ways of representing particular aspects of social life” [14]. Discourses may be used as a mechanism by which ideologies are legitimised and so help maintain domination of a subject area [15]. However, such dominance, interpreted by Fairclough as “power abuse” [16], is open to critique and CDA provides a way of identifying such dominant assumptions in order to demonstrate the implications of those assumptions [15]. In this way, CDA aims to capture the relationships between the micropolitics of the information being disseminated by both AI-funded and non-AI-funded organisations and the macropolitical landscape of power structures and ideology in which it sits [17].

In order to help uncover the dominant discourses, data were organised into themes. Dominant narratives were those deemed to carry influential messages due to the consistency and frequency with which they were mentioned across the materials. Analysis was carried out iteratively, involving continuous comparison within and between websites. This approach allowed for the discourses to be systematically identified.

Discursive themes (or “codes”; short summaries of topic areas) were constructed independently by three researchers (AR, MP and MvS), based on close reading of the data. Coding was informed by previous literature that analysed information disseminated by the alcohol industry to youth education programmes and schools using a coding framework [18]. Coding was “open” allowing for the identification of emergent codes as the analysis proceeded. The discursive elements which were identified were discussed to reach a consensus and a final coding framework was then developed (see Additional file 1).

An extraction table was developed based on the identified themes, allowing extraction of supporting quotes (see Additional file 2). The extraction table was piloted by three researchers (MvS, MP and AR) on content from four websites (two AI-funded and two non-AI-funded). One researcher (AR) then systematically analysed all information, identifying all relevant text and iteratively ascertaining any further discursive elements as generated through the analysis process. Any additional analytical elements identified through this iterative process were discussed among the researchers to reach consensus on their inclusion. AR then re-read the entire dataset, coding the data with this more comprehensive coding framework, and organising themes into overarching discourses using an inductive process as the dataset was analysed. These were discussed amongst the researchers (AR, MP, MvS), with any disagreements resolved through discussion.

### “Tone” analysis

Previous analyses looking to understand the influence of industry and other organisations on research and policy development have analysed the “tone” of articles, for example those reporting on tobacco industry-funded not-for-profit organisations [19]. We conducted a similar analysis, to assess whether the overall tone within individual websites were considered to be pro-alcohol consumption, neutral or discouraging of alcohol consumption. Two researchers (LCW and YB), who were not involved in the primary analysis, reviewed all text from the 31 websites. The text was anonymised in relation to the name and type of organisation. As with the aforementioned study that analysed the “tone” of articles related to the tobacco industry [19], the two researchers agreed upon criteria for each framing argument (the argument most frequently presented in the article), pro-alcohol consumption, neutral or against alcohol consumption, before conducting the analysis. “Pro-alcohol” was defined by a framing argument in favour of, or actively promoting, alcohol consumption, “neutral” defined by a framing argument judged not to be either actively encouraging nor discouraging consumption, “against” defined by a framing argument against alcohol consumption. A random sample of five articles was selected and coded by an additional four coders, in order to assess inter-rater reliability.

## Results

The text focusing on food consumption with alcohol consumption, and health-related outcomes, was analysed from 15 AI-funded websites and 16 non-AI-funded websites. The discourses identified in the materials are described below with example illustrative quotes.

### Food and “moderate drinking” as part of everyday life

Food and alcohol consumption were frequently discussed together in both AI-funded and non-AI-funded websites, presenting alcohol consumption alongside food consumption (an essential activity done daily) as a part of everyday life. For example, by associating alcohol with mealtimes, an alcoholic drink is sometimes portrayed (by both AI-funded and non-AI-funded organisations) as a normal part of the everyday routine, particularly with meals:

“It is better to drink wine moderately and regularly with the meals than to drink the same amount on a single occasion, and without any food.” (Wine in Moderation, AI-funded)

“On the other hand, many people enjoy the taste of alcoholic beverages. And when consumed by adults in small to moderate amounts, especially with meals, alcohol may be good for the heart.”(WebMD, non-AI-funded)

It is not clear from the accompanying text why alcohol is associated with food within these websites, and there is no evidence drawn upon or cited within the discourses to substantiate statements claiming it may be healthy “especially with meals”, as in the case of WebMD.

“Moderate” drinking, featured often in these materials. “Moderate drinking” like “responsible” drinking is a vague term that is exploited by the AI [20]. The concept of moderate drinking is subjective, inevitably conceived differently by readers, with many interpreting “moderation” to be their own current level of consumption [21]. In these documents, “moderate” alcohol consumption was often presented, almost exclusively by AI-funded organisations, as part of a healthy lifestyle along with a healthy diet and physical activity. Regular and moderate alcohol consumption was sometimes discussed in relation to the Mediterranean diet, a dietary pattern which is well-established to have health benefits [22]:

“When enjoyed in moderation, alcohol can be part of a healthy lifestyle that includes good diet and exercise.” (Beam Suntory, AI-funded)

“The moderate and daily intake of wine, usually red, during meals is also integral to the Mediterranean diet. Previous research studies have found a positive association between sticking closely to this kind of eating regimen and increasing life expectancy, as well as lowering the risk of debilitating diseases such as cardiovascular disease, type 2 diabetes, Metabolic Syndrome and dementias such as and Alzheimer’s disease” (Wine in Moderation, AI-funded)

The association of regular, moderate drinking with this diet may imply that people should consider it an integral part of a healthy diet. Discussion of longer-term harms in the extracted text is limited to CVD, where some AI-funded websites claim that alcohol consumption with food reduces cardiovascular risk. This claim represents a considerable overstatement of the evidence, as the evidence on CVD and alcohol is, at best, uncertain, despite the claim by the “Wine in Moderation” website [3, 23]. Any health benefits are uncertain given methodological and other biases in the evidence, and the epidemiological evidence suggests that any benefits from alcohol consumption, if they exist, are likely to accrue at very low levels of consumption. Additionally, they do not apply to the risk of cancer, which increases at any level of consumption [5].

### Eating to slow absorption, and to drink for longer

The concept of food playing a protective role in the consumption of alcohol, as a mechanism by which to manage alcohol consumption, was a strong discursive frame emerging from both AI and non-AI-funded websites. This frequently emphasised the need to slow down the absorption of alcohol and, in some cases, to extend the drinking “moment”. This discursive frame was seen more commonly within the AI-funded websites:

“Serving both food and drinks also makes the occasion more fun. You drink more slowly when you eat as well!” (Bacardi “slow drinking” website, AI-funded)

“In addition to enjoying the tastes, eating helps to slow down the absorption of alcohol in the blood. It’s perfect for making the moment last longer by delaying the effects of the alcohol!” (Bacardi “slow drinking” website, AI-funded)

“Eat up. Don’t drink on an empty stomach. Eat something preferably carbohydrates- before you start drinking and snack between drinks. Eating slows alcohol absorption and gives you more energy to enjoy yourself.” (Heineken website, AI-funded)

Promoting eating, as a mechanism to slow absorption of alcohol, was observed in the discourses adopted by both AI and non-AI-funded organisations. Many texts, from both types of organisation, recommended eating before consuming alcohol in order to avoid drinking on an empty stomach. Some organisations provided reasons for this, for example to delay alcohol entering the bloodstream, while others did not:

“if your child is going to drink, give them starchy food (like bread or pasta) so they won’t be drinking on an empty stomach” (Northern Ireland Direct, non-AI-funded)

“eating before you start drinking will help you the most. That’s because if you drink on an empty stomach, the alcohol will enter your bloodstream almost immediately.” (DrinkiQ (Diageo), AI-funded).

“Eat a decent meal if you’re going to be drinking alcohol- food slows down the rate that alcohol is absorbed into the bloodstream.” (Drinkaware, AI-funded)

“Pace [:] Encourage them to take their time to taste and enjoy their drinks rather than rushing or downing them.” (Drinkaware, AI-funded)

“Drinking over several hours as well as eating will have a lesser effect on your blood alcohol concentration.” (Aware.org, AI-funded)

“Eat before and while you drink [:] Eat a good meal before you start drinking, or enjoy some snacks while you drink. This helps to slow down the effect of alcohol on your body.” (Alcohol Focus [Scotland], non-AI-funded)

The need for such mechanisms to extend drinking and slow the absorption of alcohol appears in some cases to be based on the assumption that people wish to drink multiple alcoholic beverages and prolong the drinking “window”, as with the Bacardi example above. The provenance of this assertion is not discussed in such discourses, instead it is presented as a given, or an accepted norm. Slowing down the immediate effects of alcohol consumption and “making the moment last longer” may serve to increase consumption, if people are able to keep drinking steadily over longer periods of time.

### Food to prevent and cure a hangover

Food intake when consuming alcohol was also framed as a way of preventing a hangover and various websites also described the role of food as being a “cure” or a “treatment” for hangover symptoms. The majority of websites that mentioned hangovers suggested mechanisms to prevent hangover and hangover cures:

“Avoid alcohol on an empty stomach as it increases the risk of experiencing hangover symptoms. Food helps slow down the rate your body absorbs alcohol.” (Drinkaware, AI-funded)

“How can you cure a hangover?

As well as water, drink fresh juice to give yourself a vitamin boost. If you really need it, take a painkiller and an antacid to settle your stomach and alleviate hangover symptoms. Try a rehydration treatment sachet – they replace lost minerals and salt.

Eat something - bananas and kiwis are examples of food you can eat to help cure a hangover as they are a source of potassium (a mineral you lose when you drink because of the diuretic effect of alcohol).” (Drinkaware, AI-funded)

“Follow these tips to keep hangovers away:

Don’t drink on an empty stomach. Before you go out, have a meal that includes carbohydrates (such as pasta or rice) or fats. The food will help slow down the body’s absorption of alcohol.” (NHS Direct Wales, non-AI-funded)

However, there was one non-AI-funded website that discussed hangover “cures” as myth:

“Can you ‘cure’ a hangover?

Hangover cures are generally a myth. There are no cures for a hangover. All you can do is ease the symptoms and wait until it goes away.” (Health Direct [Australia], non-AI-funded)

This theme of hangover prevention was seen across both AI-funded and non-AI-funded websites and may unintentionally serve to normalise hangovers. This is despite the fact that to experience a hangover, people would likely need to be consuming alcohol well above guideline levels. While eating before or during alcohol consumption may slow the absorption of alcohol and may lower the blood alcohol concentration (BAC) at any one time [24], it will not affect the overall amount of alcohol entering the bloodstream [25]. However, it may speed up metabolization of alcohol and reduce the likelihood of a hangover, though there is surprisingly little research on this [26].

### “Responsible” alcohol consumption

“Responsible” alcohol consumption was a dominant discursive frame emerging from the analysis. “Responsible” drinking is a term almost exclusively used by industry or industry-funded organisations that is purposefully ambiguous and stresses individual responsibility, a common theme within the texts [20]. As the “Australian Guidelines to Reduce Health Risks from Drinking Alcohol” discuss, the idea of “responsible” drinking differs within different societal groups and could be considered a moral or normative standard rather than based on scientific evidence [8].

The responsibility of the individual, in terms of their behaviour while drinking, was a common theme, found almost entirely in AI-funded organisations. This was often accompanied, particularly in AI-funded websites, with a narrative relating to “control” and to the use of food to allow the individual to maintain control. Short-term negative consequences of drinking were also often emphasised in AI-funded websites as being the fault of the individual, who lacks knowledge or judgement:

“Don’t drink on an empty stomach. A healthy meal before you go out or start drinking, and snacks between drinks can help to slow down the absorption of alcohol, helping you stay in control.” (Drinkaware, AI-funded)

“Alcohol is a depressant – it can affect your reason, judgement and coordination, and slows down your reaction time. This means that if you drink to excess, you’re putting yourself at risk.” (Asahi website, AI-funded)

"Studies show that many people, especially young people and women, underestimate the amount they drink because they do not know what constitutes a standard drink." (Educ’alcool, AI-funded)

“Drinking alcohol is a matter of individual judgement and accountability.” (Asahi website, AI-funded)

“Those choosing to drink have a responsibility to get the facts about how alcohol affects them, and make smart choices when they consume alcohol.” (Beam Suntory, AI-funded)

Finally, the calorie content of alcohol is discussed on a few websites, both AI and non-AI-funded; this is not discussed further as it is not discussed in the context of consuming food as a harm reduction measure.

### “Tone” analysis

The rated tone of the materials presented on these websites was found to be similar for AI-funded websites and non-AI-funded websites (83.3% of AI-funded websites, and 81.3% of non-AI-funded websites, adopted a tone which was assessed as “pro-alcohol”). The inter-rater reliability for the random sample of five articles selected and coded by four coders equalled the threshold for an acceptable level of agreement (70.0%) [27].

## Discussion

This analysis found that there are clear differences and also some, perhaps surprising, similarities between the information on alcohol and food disseminated by AI-funded and non-AI-funded websites. There are examples of both types of organisation appearing to encourage using food as a measure to enable sustained drinking and to help avoid drinking in a way that causes *specific short-term* harms. There were also instances when both types of organisation appeared to normalise alcohol consumption and drinking to excess, as discussed below.

There were also examples of recommending food as a method of preventing or curing a hangover. Such discourses were found almost entirely on the websites of AI-funded organisations. There also appears to be an implicit assumption in some of the messages from both AI-funded and independent organisations that consumers were looking for, and would benefit from, ways to be able to drink for longer, without immediate, adverse impacts, and that consumers were likely to drink in a way that would precipitate a hangover. AI discourses also typically suggested that “moderate” drinking with food had health benefits; focused on special events such as holidays; and focused on individual responsibility. This articulation of alcohol consumption was exclusive to AI discourses.

Overall, our findings suggest that both AI and non-AI organisations frequently appear to present information in a way that normalises alcohol consumption, while framing eating as a mechanism for individuals to “manage” the effects of alcohol consumption. Here, we understand the concept of normalisation to represent processes and practices that may serve to normalise certain behaviours, ideas or values by portraying them as the “normal” or natural order of things, the “taken for granted”. Normalisation manifested in different ways, including through alignment with food, mealtimes, and in some cases the active encouragement towards consuming alcohol as part of a balanced diet.

The emphasis on “slow” drinking is a particular focus of AI websites. In this, eating is framed as making the experience of drinking more enjoyable both through enhancing taste, but also by slowing down and making the “moment last longer”. It is unclear why “slow” drinking would be a goal for consumers, however, the tension between the AI’s need to maintain and increase sales on the one hand, and to maintain its reputation on the other, may be one reason why it is promoted within AI-funded websites. Alcohol industry sales are highly dependent on people consuming alcohol to excess [28], yet industry must not be seen to perpetuate such high levels of alcohol consumption. As such, by including such “responsible drinking” guidelines on their websites, the AI are exerting a form of agenda-setting power as part of their broader CSR initiatives. It additionally allows the AI to achieve a well-used CSR technique of “innocence by association”, whereby corporations associate themselves with good causes/PR campaigns to improve their public image [29].

Promoting heavy and/or sustained drinking, while reducing visible, short-term, overt harms (e.g. being visibly drunk, being injured) is therefore a desirable strategy for the industry, i.e. through encouraging consumption of the equivalent or greater amount of alcohol, but over longer “sessions” and/or more regularly, and with food. Previous studies have demonstrated how industry seek to enact this strategy, for example, by showing how the industry frames messages of responsibility around the individual drinker and often focusses on a minority of “harmful drinkers” rather than on the majority of “moderate” drinkers [30]. Such organisations also present “responsible drinking” as a behavioural, rather than health issue—that is, the drinker simply needs to moderate their behaviour while drinking, rather than actually drink less [31]. Such discourses appear to establish a simplistic dichotomy between “good drinkers” who visibly abide by the rules, and “bad drinkers”, the minority who will not or cannot heed the advice prescribing how to “drink responsibly”.

However, these narratives, while more prevalent and explicit in the websites of AI-funded organisations, were not limited to these organisations. Concerningly, there was also frequent messaging within non-AI-funded organisations that may normalise alcohol consumption, as discussed above, for example through aligning it with food and discussing “moderation” in vague terms. We identified examples in the dataset used for this analysis of both types of website discussing the role of food in preventing or curing a hangover. This narrative may reflect an assumption that people are likely to drink in a way that will lead to a hangover, which may again contribute to normalising heavy drinking.

Specifically considering evidence of harm reduction, it is apparent that there is a lack of evidence on food, alcohol and harms cited within websites that disseminate information on the health impacts of alcohol, and even national guidelines. Given this absence, it is unclear if food consumption is an evidence-based strategy to reduce harms associated with alcohol consumption and so the justification for its uncritical dissemination may pose its own risks. Such measures might actually increase harms or merely shift the timing of harms. While prolonging a drinking session, as many webpages suggest (without supporting evidence), may reduce the risk of short-term harms such as injury or hangovers, they may be accompanied by an increase in longer-term, chronic harms, such as liver disease, due to greater overall consumption. The pervasive use of this recommendation also supports the endurance of the industry-favoured discourse structured around individual-level interventions, personal responsibility and the problematising of people not products. Given the frequency of online advice recommending food with alcohol consumption as a harm reduction measure, it is important to know if the co-consumption of food with alcohol does, in fact, reduce alcohol harms, highlighting the need for a review of the evidence.

It is also notable that the narratives around hangovers, binge drinking and encouragement of “moderate” and “slow” drinking in AI-funded materials focused to a large extent on young people and drinking. By focussing on young people, this could, again, be seen as an attempt by the industry to avoid public displays of being drunk, consistent with previous findings [32]. However, although non-AI-funded material focused more on the whole population, the existence of similar narratives around binge drinking, hangovers and “moderate” drinking may point to the concerning normalisation of excessive drinking across the board.

Strategies that focus on navigating the use of a product, rather than trying to tackle the underlying causes, have been seen across other industries. One example is in the tobacco industry, where the use of personal responsibility frames deflect attention from corporate responsibility [33], thus protecting the industry against litigation, regulation and tobacco control measures [34–36]. A similar approach is seen in the context of car-based transport systems and pedestrian safety. In the USA, for example, in response to escalating harms to pedestrians, the focus has been on blaming the behaviours of pedestrians and, to a lesser extent drivers, who need to use ever more technological advances to improve and “manage” their individual behaviour when using the transport system. Alternatives, for example to have far fewer cars on the road and significantly slow down those that remain, continue to be largely excluded from the discourse of feasible solutions [37, 38].

Among the strengths of this study are the use of discourse analysis, which offers a more critical perspective, allowing findings to be interpreted within their wider political and social context. This allows greater understanding of how specific discourse may work to promote certain ideas, ideologies and conceptualisations. Another strength is the involvement of multiple researchers in various stages of the analysis, which reduces the risk of bias. The main weakness is that only website material was included—both public health bodies and AI-funded organisations have other means of communication that would also benefit from analysis (such as social media).

## Conclusions

Our findings add to the evidence that alcohol consumption, including heavy and harmful consumption, is normalised within the online information space. Discussing harmful alcohol consumption in the context of food may contribute to this normalisation. It is essential that all online sources of information on these topics consider whether they are promoting pro-alcohol, pro-consumption narratives, inadvertently or not. The fact that both AI-funded and non-AI-funded organisations shared many of these narratives requires further analysis.

From a policy perspective, health information sources that are assumed to be trustworthy and evidence-based, such as national guidelines, should ensure harm reduction messaging is informed by the best available evidence, and free from the influence of actors with conflicts of interests (e.g. commercial or financial). A more immediate harm reduction strategy could involve the development and propagation of a counter-narrative to the dominant framing of alcohol and food. This counter-narrative would act as a harm reduction message in of itself, to increase awareness and inform public health actors, decision-makers, and the public at large, of the prevailing industry tactics and highlight actual evidence-based alcohol-related harm reduction measures. Furthermore, independent health professionals and independent health organisations should review the information they provide in light of our findings and continually challenge why alcohol industry-funded organisations with a major conflict of interest, and a history of health misinformation, are given the responsibility for disseminating health information to the public in the first place.

## Supplementary Information

**Additional file 1** Codes identified by researchers, iteratively grouped into themes and dominant discourses following discussion.

**Additional file 2** Extraction table sample used in analysis, with three examples from AI-funded organisations and three from non-AI-funded organisations.

## Data Availability

Not applicable.
