# Supplementary material for: Food as harm reduction during a drinking session: reducing the harm or normalising harmful use of alcohol? A qualitative comparative analysis of alcohol industry and non-alcohol industry-funded guidance
Source: Harm Reduct J. 2022 Jun 25;19:66. doi: 10.1186/s12954-022-00648-y (PMC9233813; doi:10.1186/s12954-022-00648-y)
Supplement: Supplementary file 1 — Additional file 1 Codes identified by researchers, iteratively grouped into themes and dominant discourses following discussion. [file 12954_2022_648_MOESM1_ESM.docx]

**Additional File 1. Codes identified by researchers, iteratively grouped into themes and dominant discourses following discussion**

| **Coder** | **Code** | **Theme** | **Dominant Discourse** |
| --- | --- | --- | --- |
| MVS | Drinking as a part of the diet | Normalisation of alcohol through alignment with food | Food and "moderate drinking" as part of everyday life |
| MVS | Aligning alcohol and drinking with diet / food – normalising it | Normalisation of alcohol through alignment with food |  |
| MVS | Alcohol is just like any other shopping trolley item | Normalisation of alcohol through alignment with food |  |
| MVS | Can be “incorporated into the calorie limits of most healthy eating patterns” | Normalisation of alcohol through alignment with food |  |
| MVS | Aligning with Mediterranean diet | Normalisation of alcohol through alignment with food |  |
| AR | Lumping alcohol together with food and drink more generally | Normalisation of alcohol through alignment with food |  |
| AR | Normalisation of drinking alcohol, reassuring, part of a majority – everybody does it | Normalisation of alcohol through alignment with food |  |
| AR | Language that makes drinking the inevitable and the default. Not ‘if’ but ‘when’ | Normalisation of alcohol through alignment with food |  |
| MP | Moderation | What is moderation |  |
| MVS | Moderation versus excessive | What is moderation |  |
| MVS | Moderation versus excessive | What is moderation |  |
| AR | Vague definitions, such as drinking, moderation, excessive | What is moderation |  |
| MP | Food and drink as a positive RF (“Daily moderate drinking as part of a high-quality diet”) | Moderate drinking for health |  |
| MP | There is a certain level of drinking that is good for you. Baseline level of drinking that is safest- i.e. j-shaped curve. | Moderate drinking for health |  |
| AR | Low-risk drinking as the healthiest option without mention of no drinking. | Moderate drinking for health |  |
| MP | Pacing to maintain drinking over a longer period | Increasing capacity to drink more e.g. pacing | Eating to slow absorption, and to drink for longer |
| AR | Pacing, drinking slowly, eating to slow absorption, counting drinks - pacing | Increasing capacity to drink more e.g. pacing |  |
| AR | Eating as part of the strategy to slow drinking, pacing, spreading it out, drinking properly | Increasing capacity to drink more e.g. pacing |  |
| AR | Eating to increase energy | Increasing capacity to drink more e.g. pacing |  |
| MP | Slow down or pace the drinking and “have more fun” | Reason for recommending food |  |
| MP | Eat before you drink | Reason for recommending food |  |
| MP | Slows absorption | Reason for recommending food |  |
| MP | Eating food will not stop you getting drunk | Reason for recommending food |  |
| MVS | The mechanism | Reason for recommending food |  |
| MP | Food reduces risk of hangover | Food to prevent/ cure hangover | Food to prevent and cure a hangover |
| MP | Food to cure a hangover | Food to prevent/ cure hangover |  |
| MVS | The role of food in avoiding or treating a hangover | Food to prevent/ cure hangover |  |
| AR | Hangover as something to be cured or treated with food–reducing the perception of excessive alcohol drinking as a health risk. Food as a prevention, cure or treatment | Food to prevent/ cure hangover |  |
| AR | Including specific hangover symptoms – creates image and makes it more “real” | Food to prevent/ cure hangover |  |
| MP | Control | Control | "Responsible" alcohol consumption |
| MP | Individual-blaming | Control |  |
| MVS | Controlling how fast you absorb alcohol by using food is important | Control |  |
