# Supplementary material for: Food as harm reduction during a drinking session: reducing the harm or normalising harmful use of alcohol? A qualitative comparative analysis of alcohol industry and non-alcohol industry-funded guidance
Source: Harm Reduct J. 2022 Jun 25;19:66. doi: 10.1186/s12954-022-00648-y (PMC9233813; doi:10.1186/s12954-022-00648-y)
Supplement: Supplementary file 2 — Additional file 2 Extraction table sample used in analysis, with three examples from AI-funded organisations and three from non-AI-funded organisations. [file 12954_2022_648_MOESM2_ESM.docx]

**Additional File 1. Sample extraction table with examples from three AI-funded organisations and three non-AI-funded organisations**

| **Extracted by** | **Organisation type** | **Organisation name** | **Theme** | **Supporting quote - free text** |
| --- | --- | --- | --- | --- |
| AR | AI-funded | Aware.org (South Africa) | Increasing capacity to drink more e.g. pacing | "Drinking over several hours as well as eating will have a lesser effect on your blood alcohol concentration." |
| AR | AI-funded | Aware.org (South Africa) | Behavioural consequences | "Binge drinking can lead to anti-social and aggressive behaviour which could have detrimental impacts on relations with loved ones and friends." |
| AR | AI-funded | Aware.org (South Africa) | Type of drinking | "Binge-drinking can have disastrous consequences on your health and wellbeing." |
| AR | AI-funded | Aware.org (South Africa) | Increasing capacity to drink more e.g. pacing | "Drink more slowly and try to alternate each drink with a non-alcoholic beverage and food." |
| AR | AI-funded | Aware.org (South Africa) | Control | "Avoid risky people and places. Always ensure that you are with people that you know and make sure that you have a plan to get home safely." |
| AR | AI-funded | Aware.org (South Africa) | Individual/ circumstantial variation | "Women are generally affected more quickly by alcohol and by smaller amounts than men which means they should drink slower and less than their male counterparts." |
| AR | AI-funded | Aware.org (South Africa) | Type of drinking | "Some of us aren’t sure if we’re still within safe drinking parameters; even those of us who drink more than the recommended 14 units a week." |
| AR | AI-funded | Aware.org (South Africa) | Drinking as part of socializing and celebrating | "For some people, a drink or two after clocking off for the weekend or for a holiday marks the start of the fun and relaxation." |
| AR | AI-funded | Aware.org (South Africa) | Control | "People vary in whether they get hangover – some spoil their precious time off in that way." |
| AR | AI-funded | Aware.org (South Africa) | Drinking as part of socializing and celebrating | "If you want to toast your time off" |
| AR | AI-funded | Aware.org (South Africa) | Normalisation of alcohol through alignment with food | "Why not go to a restaurant rather than the pub? That way you can have something to eat and make sure that the evening revolves around food rather than alcohol." |
| AR | AI-funded | Educ'alcool | Type of drinking | "Alcohol is a normal part of life for most of us, and fortunately, it is generally not problematic. In Quebec in particular, moderation has become both socially integrated and culturally acceptable." |
| AR | AI-funded | Educ'alcool | Individual/ circumstantial variation | "The limits are different because research shows that alcohol puts women at greater risk for certain alcohol-related illnesses than men (e.g., breast cancer, stroke, diabetes, high blood pressure, liver disease)." |
| AR | AI-funded | Educ'alcool | Individual/ circumstantial variation | "These gender differences occur for several reasons." |
| AR | AI-funded | Educ'alcool | Moderate drinking for health | "While alcohol may be of some benefit to post-menopausal women and men over the age of 40" |
| AR | AI-funded | Educ'alcool | Increasing capacity to drink more e.g. pacing | "Tips:  • Set limits for yourself and stick to them. • Drink slowly. Have no more than 2 drinks in any three-hour period. • Alternate with non-alcoholic drinks. • Eat before and while you are drinking. • Plan to drink in a safe environment. • Know your local laws." |
| AR | AI-funded | Educ'alcool | Individual/ circumstantial variation | "However, as the research shows with increasing clarity, when it comes to alcohol, we are not all born equal." |
| AR | AI-funded | Educ'alcool | What is moderation? | "Genetic heritage, weight and age are among the important factors we must all consider when figuring out what “moderate drinking” means for us." |
| AR | AI-funded | Educ'alcool | Individual/ circumstantial variation | "Genetic heritage, weight and age are among the important factors we must all consider when figuring out what ‘moderate drinking’ means for us." |
| AR | AI-funded | Educ'alcool | Control | "Studies show that many people, especially young people and women, underestimate the amount they drink because they do not know what constitutes a standard drink." |
| AR | AI-funded | Wine in Moderation (www.wineinmoderation.eu) | Moderate drinking for health | “The moderate and daily intake of wine, usually red, during meals is also integral to the Mediterranean diet.  Previous research studies have found a positive association between sticking closely to this kind of eating regimen and increasing life expectancy, as well as lowering the risk of debilitating diseases such as cardiovascular disease, type 2 diabetes, Metabolic Syndrome and dementias such as and Alzheimer’s disease” |
| AR | AI-funded | Wine in Moderation (www.wineinmoderation.eu) | Moderate drinking for health | “Regular moderate wine consumption has been associated with certain potential health benefits for adults, particularly those aged over 40 years” |
| AR | AI-funded | Wine in Moderation (www.wineinmoderation.eu) | Normalisation of alcohol through alignment with food | “It is better to drink wine moderately and regularly with the meals than to drink the same amount on a single occasion, and without any food.” |
| AR | AI-funded | Wine in Moderation (www.wineinmoderation.eu) | Moderate drinking for health | “Regular moderate wine consumption has been associated with several health benefits. However the risk increases with each drink above moderation!” |
| AR | AI-funded | Wine in Moderation (www.wineinmoderation.eu) | Moderate drinking for health | “Moderate wine drinkers have a lower mortality rate than those who abstain or drink heavily. This widely accepted association is known as the J-curve. The relative risk of dying is lowest among light to moderate drinkers and greater among abstainers” |
| AR | AI-funded | Wine in Moderation (www.wineinmoderation.eu) | Type of drinking | “The authors concluded that it is best for drinkers to avoid binge drinking -- not only because of the possible heart effects, but also because of more immediate risks, like accidents and violence.” |
| AR | Government/non-AI funded | Northern Ireland Direct (www.nidirect.gov.uk) | "Responsible" drinking | "Promoting a responsible attitude to alcohol  Parents and carers have an important role in showing their children a responsible attitude to alcohol" |
| AR | Government/non-AI funded | Northern Ireland Direct (www.nidirect.gov.uk) | Reason for recommending food | "if your child is going to drink, give them starchy food (like bread or pasta) so they won’t be drinking on an empty stomach" |
| AR | Government/non-AI funded | Northern Ireland Direct (www.nidirect.gov.uk) | Individual/ circumstantial variation | "Medical guidelines advise men and women who wish to keep their short term health risks from a single drinking occasion to a low level that they can reduce these risks by: • limiting the total amount of alcohol you drink on any occasion • drinking alcohol more slowly • eating food while drinking alcohol • alternating alcoholic drinks with glasses of water" |
| AR | Government/non-AI funded | Northern Ireland Direct (www.nidirect.gov.uk) | Individual/ circumstantial variation | "These guidelines are for adults. As young people take longer to process alcohol, it is a good idea for them to drink less, or not to drink at all." |
| AR | Government/non-AI funded | Northern Ireland Direct (www.nidirect.gov.uk) | Control | "One in ten young people who drink alcohol end up in trouble with the police. They can get involved in anti-social or criminal behaviour" |
| AR | Government/non-AI funded | Northern Ireland Direct (www.nidirect.gov.uk) | Individual/ circumstantial variation | "Your bloodstream distributes alcohol quickly to your brain, kidney, lungs and liver. On average, your liver takes an hour to break down one unit of alcohol. This can depend on: • Your weight • Your gender • Your age • How quickly your body turns food into energy • How much food you’ve eaten • The strength and type of alcohol • Any medicine you’re taking" |
| AR | Government/non-AI funded | Northern Ireland Direct (www.nidirect.gov.uk) | Reason for recommending food | "Drinking with an empty or full stomach If you drink alcohol with an empty stomach, the alcohol passes directly into your bloodstream. If you’ve eaten before drinking, the rate of alcohol absorption slows but doesn’t stop." |
| AR | Government/non-AI funded | WebMD (USA) | Normalisation of alcohol through alignment with food | “On the other hand, many people enjoy the taste of alcoholic beverages. And when consumed by adults in small to moderate amounts, especially with meals, alcohol may be good for the heart.” |
| AR | Government/non-AI funded | Health Direct (www.healthdirect.gov.au) | Individual/ circumstantial variation | “Although the rate of absorption can differ depending on a number of factors, including sex, body size and composition, age, experience of drinking, genetics, nutrition and individual metabolism, it generally only takes a few minutes for alcohol to reach the brain.” |
| AR | Government/non-AI funded | Health Direct (www.healthdirect.gov.au) | What is moderation? | “What is a standard drink? Working out exactly what is a standard drink is not always easy. However, one standard drink contains 10g of pure alcohol (equivalent to 12.5ml of pure alcohol)” |
| AR | Government/non-AI funded | Health Direct (www.healthdirect.gov.au) | What is moderation? | “If you are a healthy adult, and limit your drinking to no more than 2 standard drinks on any one day, you will reduce your risk of harm from alcohol-related disease or injury to a low level.” |
| AR | Government/non-AI funded | Health Direct (www.healthdirect.gov.au) | Individual/ circumstantial variation | “A younger person’s body doesn’t cope with alcohol as well as an older person’s. The younger person’s brain, heart and liver aren’t fully developed and so they are less able to process it. Alcohol can therefore seriously damage a younger person’s health.” |
| AR | Government/non-AI funded | Health Direct (www.healthdirect.gov.au) | "Responsible" drinking | “Tips on how to drink responsibly • Keep an eye on what you’re drinking; set limits for yourself and stick to them. • Start with non-alcoholic drinks and alternate with alcoholic drinks, or try drinks with a lower alcohol content. • Eat before or while you are drinking. • Don’t drink and drive. • If you are going out in a group, work out who will drive everyone home. If no one wants to be the nominated driver, bring enough money for a taxi. • Avoid mixing alcohol and medication. • Understand that your blood alcohol will continue to rise after you have consumed your last drink. You generally won’t reach your maximum BAC until 45-90 minutes after consuming your final drink.” |
| AR | Government/non-AI funded | Health Direct (www.healthdirect.gov.au) | Food to prevent/cure hangover | “Can you ‘cure’ a hangover? Hangover cures are generally a myth. There are no cures for a hangover. All you can do is ease the symptoms and wait until it goes away.”  “Drink water to treat dehydration. Don’t drink any more alcohol – it will make you feel worse. Try to eat some simple food to boost your blood sugar and settle your stomach.” |
